# UJAmI Location: A Fuzzy Indoor Location System for the Elderly

**DOI:** 10.3390/ijerph18168326

**Published:** 2021-08-06

**Authors:** Antonio-Pedro Albín-Rodríguez, Yolanda-María De-La-Fuente-Robles, José-Luis López-Ruiz, Ángeles Verdejo-Espinosa, Macarena Espinilla Estévez

**Affiliations:** 1 Education and Sports Council, Junta de Andalucía (Regional Government of Andalusia), 23007 Jaén, Spain; 2 Social Work Department, University of Jaén, 23071 Jaén, Spain; ymfuente@ujaen.es; 3 Department of Computer Science, University of Jaén, 23071 Jaén, Spain; llopez@ujaen.es (J.-L.L.-R.); mestevez@ujaen.es (M.E.E.); 4 Electrical Engineering Department, University of Jaén, 23071 Jaén, Spain; mverdejo@ujaen.es

**Keywords:** bluetooth low energy, beacons, ageing people, fuzzy logic, received signal strength indicators, indoor location system

## Abstract

Due to the large number of elderly people with physical and cognitive issues, there is a strong need to provide indoor location systems that help caregivers monitor as many people as possible and with the best quality possible. In this paper, a fuzzy indoor location methodology is proposed in a smart environment based on mobile devices and Bluetooth Low Energy (BLE) beacons where a set of Received Signal Strength Indicators (RSSI) is received by mobile devices worn by the inhabitants. The use of fuzzy logic and a fuzzy linguistic approach is proposed to deal with the imprecise nature of the RSSI values, which are influenced by external factors such as radio waves, causing significant fluctuations. A case study carried out at the Smart Lab of the University of Jaén (UJAmI Smart Lab) is presented to demonstrate the effectiveness of the proposed methodology, where our proposal is compared with a non-fuzzy logic approach, obtaining an accuracy of 91.63%, approximately 10 points higher than the methodology without using fuzzy logic. Finally, our theoretical proposal is accompanied by a description of the UJAmI Location system, which applies the theory to the functionality of locating elderly people in indoor environments.

## 1. Introduction

Currently, life expectancy is above 80 years due to improved quality of life, which means that the number of older people worldwide is growing rapidly. In 2020, the number of people over 65 years of age was 727 million (9.3% of the total world population), and it is estimated that the population of elderly people will double to 1.5 billion (16.0% of the total world population) over the next three decades [1].

People are ageing and many have a strong need to stay in their homes, even if they live alone, which means that systems are needed to monitor their behaviour in order to anticipate or alert to undesired situations [2]. Indoor positioning systems (IPSs) represent a key tool for behavioural monitoring [3] because they make it possible to monitor relevant behavioural habits in people, which can be used as an indicator of falls [4] or for the recognition of human activities [5,6]. This technology can provide us with information, for example, on whether an inhabitant has been in the kitchen eating, has spent too much time on the sofa, waking and sleeping times or the number of times he/she has visited the bathroom.

One of the main challenges of IPSs when wanting to accurately estimate location is dealing with the uncertainty inherent to the applied technologies in these systems [6,7,8] due to calibration issues, data loss, indoor obstacles or battery consumption limitations. In addition, there is a significant gap between the number of theoretical proposals in the literature and those that are developed in real systems for real-life applications.

To provide solutions to these two challenges, this paper presents two proposals, one theoretical and one practical: First, a fuzzy indoor location methodology based on BLE beacons is presented in order to address the uncertainty involved in the location process. Second, the fully-functional UJAmI Location system developed on the basis of the proposed methodology is presented.

Regarding the theoretical proposal, a fuzzy indoor location methodology with the use of fuzzy linguistic terms and fuzzy temporal windows to manage the fluctuations of the BLE beacons has been proposed. These approaches have provided excellent results in other contexts with uncertainty present in sensor data, such as activity recognition [6,9,10], pressure ulcers [11], preeclampsia [12] or cardiology [13,14]. Furthermore, to validate the theoretical proposal, a case study is carried out at the UJAmI Smart Lab [15] of the University of Jaén using the UCAmI Cup dataset [16], which is available for download online https://ceatic.ujaen.es/ujami/en/repository (accessed on 5 August 2021).

To achieve the practical proposal, the indoor location system UJAmI Location has been developed under the proposed methodology. This application has been developed with the aim of providing a real solution to the previously discussed challenges that affect ageing populations. The functionality of this system is presented through case studies in the context of the UCAmI Cup dataset.

This paper is organised as follows. In Section 2, related works based on indoor location are reviewed in the ageing context. In Section 3, the fuzzy indoor location methodology is proposed. A case study to validate the proposed methodology is presented in Section 4. The UJAmI Location system implementing the proposed fuzzy methodology is then described in Section 5. In Section 6, a discussion of the proposals presented in this paper is addressed. Finally, conclusions are drawn in Section 7.

## 2. Indoor Location in the Context of Ageing

In this section, we describe the importance of indoor location as a relevant current topic.

**Figure 1:**
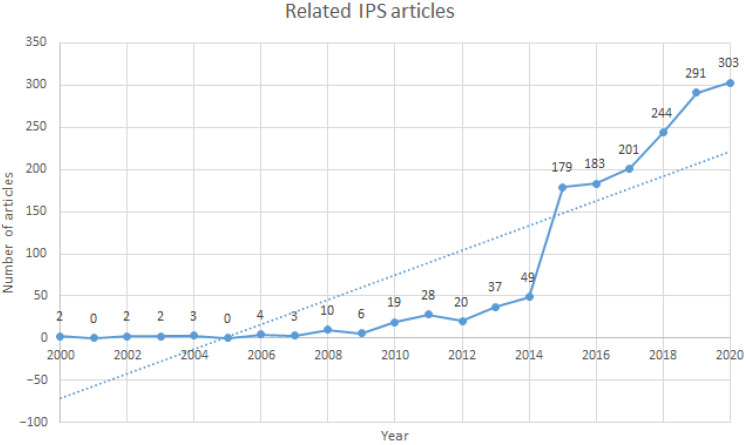
Works related to IPS between 2000 and 2020.

In the context of ageing populations, there is a special need for indoor location solutions in multiple scenarios. For example, detecting the location of a resident in a nursing home at all times while checking what inhabitants are doing and whether anything unusual is happening. In response to this demand, monitoring systems based on the Internet of Things (IoT) have emerged. These spaces are also referred to as smart homes and are composed of smart devices that are often unobtrusive in ambient-assisted living contexts [10]. This fact has attracted the attention of numerous researchers over the last two decades. This is demonstrated by the fact that when filtering by “Indoor Location System” and “Positioning Location System” in the Scopus platform, approximately 1500 papers are retrieved. The increasing trend in the number of papers related to this topic between 2000 and 2020 is illustrated in Figure 1.

There is a multitude of real-life applications for IPSs. For example, as we mentioned, identifying ways of monitoring aging populations [5,6,10]. Another example is their application in the retail sector: knowing where customers are at any given time, their path can be analysed for commercial purposes [17]. Furthermore, these systems can be used to guide customers in a shop and facilitate the search for items [18]. Finally, another example is their use in emergency situations, such as behavioural analysis of trajectory in drills [19], which can help improve indoor evacuations, or even in orientation in enclosed and poorly lit spaces such as may be the case in subway tunnels [20].

A relevant definition of IPS is provided by Brena et al. [3], who consider it as the estimation of the target’s location from the input data collected from a set of sensors. In many cases, these location systems perform the estimation using two methods: a reference to a specific location, e.g., “bedroom”, or a position based on coordinates. The general aim of IPSs is to obtain the position of users or objects, but the way in which this is achieved differs depending on the technology used. Yang et al. [21] identify a location system as a set of beacons and a sensor associated with the target that allows the user to be located by processing wireless signals [22].

Multiple approaches or models that provide methodologies based on different technologies to locate people within enclosed spaces have been proposed. In this context, it is very common to use Ultra-Wideband [6,23,24], BLE combined with a device that has Bluetooth connection (activity band or other) [25,26,27,28] or even Radio-Frequency Identification [29,30,31,32].

This paper proposes the use of BLE transmitters or beacons for indoor location [33] due to the fact that these devices are widely used and are known for their excellent performance in terms of battery, small size, light weight, high accuracy for positioning and, finally, for being easily deployable at a low cost. BLE technology emerged in 2009 designed for IoT as an extension of Bluetooth Classic [34]. In this case, the technology is intended for cases where it is not necessary to exchange a lot of data continuously. This means very low power consumption compared to the previous version, improving their characteristics: increased range, more secure connections and greater packet capacity. BLE versions 5.0 and 5.1 are the most recent versions and again enhance the features mentioned above [35,36]. In addition, version 5.1 adds Angle of Arrival and Angle of Departure, which provide a new form of location accuracy. The latest version of BLE is 5.2, and it substantially improves the technology with a new Enhanced Attribute Protocol that improves performance and speed when multiple devices are connected simultaneously, less power consumption, reduced interference with other devices and improved connection reliability [37].

In the literature, multiple Bluetooth-based indoor location systems have been proposed to obtain location based on the following six main parameters: proximity, triangulation, centroids, radio signal strength (RSS), fingerprinting or hybrid techniques combining the previous ones.

The first one of these is proximity-based. Proximity [38,39] and laterality [40] techniques were already being used in the 2000s, but a long time was needed between scans. It is a very simple technique that relies on the proximity of the target to the highest RSS value, though this is not entirely true under non-line-of-sight propagation. If we know where an object is located and a user approaches the object, then we know which area it is in [41]. The main problem with this technique is that it does not provide very high accuracy, requiring the use of different position calculation techniques [42]. An early work in this field was done by Faragher and Harle [26]. It showed a comparison between WiFi and BLE technology. The authors observed one of the most important challenges in BLE positioning: fast fading, more noticeable even than in WiFi technology. The second method we found is multilateration. This positioning technique is based on triangulation, using the distance between the target to be located and the beacons to estimate its position [43]. The main measures used to perform this estimation are Time of Flight, Time Difference of Arrival and propagation loss. The next technique is based on Bluetooth RSSI values and utilises a propagation model to estimate distance based on path loss. Examples of this type of method are Zhu et al. [44] and Neburja et al. [45]. Finally, there is the fingerprint-based positioning technique. This method is very popular and is not only used with BLE. It mainly consists of two phases: calibration and positioning. The calibration phase, also called training phase or offline phase, aims to collect the signal strength from different beacons positioned at reference points (RP) and each RP has a signal pattern or fingerprint. In the training phase, it is essential to filter out erroneous values, because RSSI values fluctuate over time [46]. This approach has been very important for BLE positioning. Fingerprint is an excellent choice for proximity if the number of beacons is small, as shown in Mendoza et al. [42].

The revised approaches provide differing accuracy, depending on the number of beacons used, the environment and the size of the site. Mendoza et al. [47] provided a review of these methods, showing that accuracy varies between one and three metres. More than half of these experiments were in small environments (between 12 m${}^{2}$ and 176 m${}^{2}$), and the remaining were in much larger spaces (an entire floor or several offices). The results have shown that location experiments in larger environments have higher accuracy.

With Bluetooth technology, decisions can be made on how to deploy the beacons according to the purpose of the positioning. However, it is a challenge to find a combination of beacons for every environment. Other challenges to be considered are the fast fading problem discussed above, and other issues such as multi-path and the absorption of the human body at 2.4 GHz frequencies [48], which is particularly evident in smartwatch devices [49]. Furthermore, the environments themselves can often restrict beacon placement, and in some cases a thorough search of the parameter space is unfeasible [50]. In this paper, we have considered that the best option is to deploy the beacons in an indoor space and to reduce positioning errors through parameters such as temporal window size, aggregation method, sampling frequency and transmit power.

Energy costs are a further factor to be taken into account in this type of system. Like in smart cities, it is always beneficial to have a minimum cost for real-time location and energy efficiency at all times [51] due to the fact that the power consumption of BLE beacons is an indispensable challenge.

Regarding the data privacy and security of IPSs, many of them, including the one proposed in this paper, use mobile devices that include built-in sensors to obtain accurate location data so as to inform about physical activity level and mental health. The study provided in [52] showed that data could be easily accessible when using mobile devices, and that users are unaware of the dangers involved and have a false sense of privacy. There is no unified solution to cover all threats in mobile technology security. However, a further fundamental consideration is that any system based on sensor data from mobile technology ensures better privacy than systems based on vision cameras [53]. The security and privacy considerations of the system proposed in our paper will be discussed in Section 6.

## 3. Fuzzy Indoor Location Methodology

In this paper, we present an indoor location methodology based on BLE technology using fuzzy logic techniques to deal with the uncertainty associated with technology-derived problems. To do so, first, a fuzzy framework is proposed, followed by fuzzy aggregation for indoor location.

### 3.1. Fuzzy Framework for Indoor Location

The system proposed in this paper is based on proximity positioning in the enclosed space, which is calculated taking into account the signal emitted by the beacon and the signal that the mobile device is able to receive (RSSI).

The following notions and terminology areas are presented in the proposed fuzzy framework using mobile devices.

(1) A smart environment in which a set of area classes exist is defined as $\left\{A^{1},\ldots ,A^{l},\ldots ,A^{L}\right\}$.
(2) There is a set of BLE beacons $\left\{B^{1},\ldots ,B^{j},\ldots ,B^{J}\right\}\in A^{l}$ that is associated with a unique area or in an object of this area, $A^{l}$.
(3) Each inhabitant has an associated mobile device *D*. The mobile device can be a smartphone, smartwatch or wearable device, the only requirement is that it receives the RSSI value provided by the BLE beacons deployed in the smart environment. Each device receives the RSSI value reading frequency, and these readings generate the RSSI signal stream $St_{RSSI}$. In this work, the stream is defined by a set of measures $St_{RSSI}=\left\{m_{i}\right\}$ where each measure is defined by a 3-tuple $m_{i}=\left\{B_{i}^{j},v_{i},t_{i}\right\}$, where $B_{i}^{j}$ is the beacon that has an associated area $A^{l}$, and $v_{i}$ is the RSSI value of this beacon in a timestamp $t_{i}$.
(4) A fuzzy linguistic term called proximity *P* is defined with membership function $\mu_{p}\left(x\right)$, being $V_{m_{i}}=\mu_{p}\left(v_{i}\right)\in \left[0,1\right]$ the membership degree of $v_{i}$ in $m_{i}$ contained in RSSI for the linguistic term *P* [54].
(5) A fuzzy temporal window, TW, is defined with membership function $T_{m_{i}}=\mu_{TW}\left(\Delta t_{i}\right)$∈[0,1] [54], where $\Delta t_{i}$ is described directly as a distance function of each sample timestamp to the current time $\Delta t_{i}=t_{i}-t_{0}$.

### 3.2. Fuzzy Aggregation for Indoor Location

Based on previous works [6,9,10,11,14], we have integrated fuzzy aggregation of the terms in the RSSI stream using a fuzzy temporal window.

First, proximity membership degrees, $V_{m_{i}}$, for each $v_{i}$ in the RSSI signal stream RSSI are computed with its fuzzy temporal window $T_{m_{i}}$ by Equation (1).

(1) $$V_{m_{i}}\cap T_{m_{i}}=\mu_{p}\left(v_{i}\right)\cap \mu_{TW}\left(\Delta t_{i}\right)\in \left[0,1\right];for\;each\;m_{i}\in St_{RSSI}$$

A joined set per area $BA^{l}$ is defined for all the measures of the $St_{RSSI}$ that includes the aggregated membership degrees related with beacons of the same area by Equation (2).

(2) $$BA^{l}=\underset{B^{j}\in A^{l}}{⋃}V_{m_{i}}\cap T_{m_{i}}\in \left[0,1\right];where\;\left\{B^{1},\ldots ,B^{j},\ldots ,B^{J}\right\}\in A^{l}$$

We note that several fuzzy operators can be used to implement the aggregation. However, in this paper, we propose a fuzzy weighted average [54], which is recommended in cases where there are high sample rates from sensors [6]. The aggregation process is defined by Equation (3).

(3) $$V_{m_{i}}\cap T_{m_{i}}=\frac{\sum_{}V_{m_{i}}\times T_{m_{i}}}{\sum T_{m_{i}}}$$

The area corresponding to the maximum value of the fuzzy aggregation of proximity values per area in a fuzzy temporal window is assigned to the timestamp $t_{i}$ by Equation (4).

(4) $$Loc_{t_{i}}=Max\left(BA^{l}\right);l\left\{1,\ldots ,L\right\}$$

## 4. Case Study

In this section, we describe a case study in order to properly evaluate the effectiveness of the proposed methodology presented in Section 3.

To do so, we describe the selected dataset and the details of the environment in which the data were collected. Then, we explain the processing that was applied using the proposed fuzzy methodology to obtain the person’s indoor location. Finally, a comparison between the methodology with fuzzy processing and without fuzzy processing is performed.

### 4.1. UCAmI Cup Dataset

The research study has been carried out at the UJAmI Smart Lab [15] of the University of Jaén by using the UCAmI Cup dataset [16,55]

This smart lab is a small intelligent apartment divided into several areas: a living room, a dining room, a bathroom, a bedroom and a kitchen. These areas can be used by one or more inhabitants at the same time. The bedroom is integrated with the bathroom (toilet and sink). The kitchen is very large and has plenty of storage space. It also includes basic appliances such as a washing machine, dishwasher, oven and microwave. In addition, the smart lab includes a living room with a sofa, a television and a work space which can be considered to be an integrated office.

**Figure 2:**
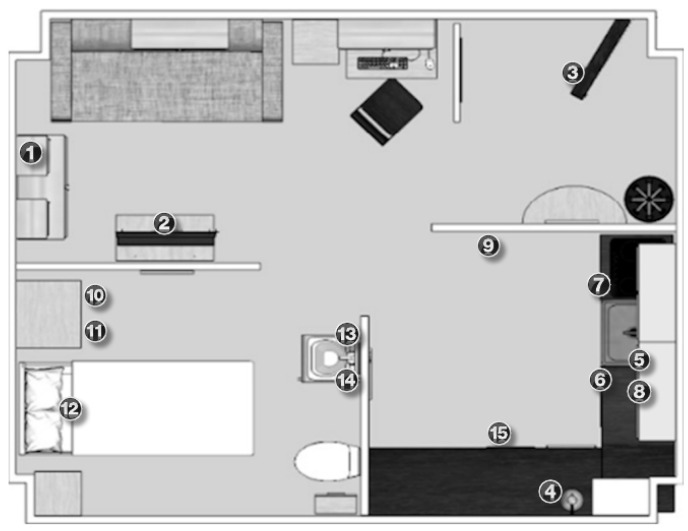
Location of Estimote Sticker beacons in the UJAmI Smart Lab. (**1**) TV controller, (**2**) Book, (**3**) Entrance door, (**4**) Medicine box, (**5**) Food cupboard, (**6**) Fridge, (**7**) Pot drawer, (**8**) Water bottle, (**9**) Garbage, (**10**) Wardrobe door, (**11**) Pyjamas drawer, (**12**) Bed, (**13**) Bathroom tap, (**14**) Toothbrush, (**15**) Laundry basket.

To validate the proposed methodology, the UCAmI Cup dataset [16,55] was used to obtain particular fingerprints or location patterns. The dataset was generated by a person over a period of 10 days by obtaining data from four heterogeneous sources located in the UJAmI Smart Lab. Among them, there is proximity information between a mobile device and 15 BLE beacons placed on various objects in the smart lab. These beacons were fixed in all areas of interest in each of the smart lab areas. The placement of the beacons for this case study is shown in Figure 2.

The data were collected each day and were divided into three subsets corresponding to morning, afternoon and evening, each subset with an approximate duration of 90 min. In addition, the dataset contains the activities that the inhabitant carried out during the data acquisition. As our work focuses on indoor location, we have associated each activity with the area or areas in which it is performed. For example, the activity *Go to bed* has been associated with the bedroom area. Table 1 shows the correspondence between the activities and the smart lab areas.

### 4.2. Intelligent Processing Using the Fuzzy Indoor Location Methodology

In this subsection, the proposed fuzzy indoor location methodology presented in Section 3 is applied in the UCAmI Cup dataset to process the location of the inhabitant in an intelligent way.

This application of the methodology uses, on the one hand, the proximity data source obtained from BLE beacons and a mobile app installed on a device and, on the other hand, the data of the activities that the inhabitant carried out during data acquisition. The proximity data stored in the dataset contains the following information: timestamp, unique identifier of the beacon, object with which the beacon is associated and, finally, the collected RSSI value.

**Figure 3:**
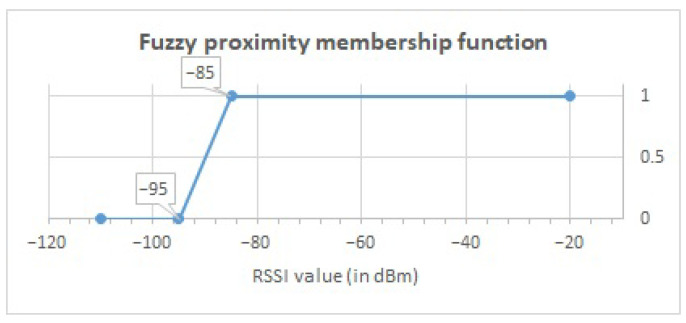
Trapezoidal membership functions for proximity value.

**Figure 4:**
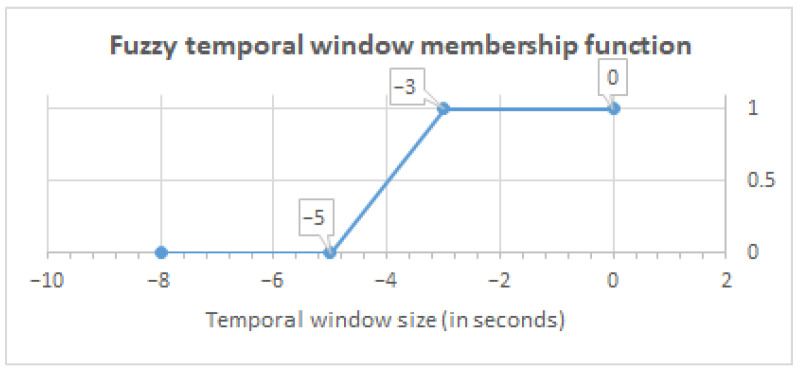
Trapezoidal membership functions for temporal window.

In order to carry out the validation of the proposed fuzzy methodology presented in Section 3, the dataset was processed based on the following parameters: (1) A fuzzy proximity value defined by the trapezoidal membership function and (2) a fuzzy temporal window size defined by the trapezoidal membership function. These functions are illustrated in Figure 3 and Figure 4.

To explain the intelligent processing with the proposed fuzzy methodology, a fragment of the UCAmI dataset has been selected (see Table 2), corresponding to the *Brush teeth* activity carried out by the inhabitant.

As can be observed, this subset provides the following information:

- Temporal Window (TW) represents the temporal window identifier to manage the fluctuations of the RSSI values from BLE beacons. The most accurate correlation between the activity carried out and the inhabitant’s location is obtained using a 5-second temporal window.
- Datetime (DT) is the date and time at which the RSSI value of the beacon was obtained by the mobile device.
- Beacon (Bc) shows the name of the beacon from which the RSSI value has been obtained by the mobile device.
- Beacon Area (Bc Area) indicates the area where the beacon is located in the smart lab, as illustrated in Figure 2.
- RSSI is the value received in the mobile device for the signal emitted by the beacon.
- Fuzzy Value represents the fuzzy RSSI value obtained from the fuzzy proximity membership function proposed in Figure 3.
- Bathroom area (BTA) specifies the calculated fuzzy values grouped in the defined temporal window, after applying the membership function proposed in Figure 4, and that belong to beacons located in the bathroom area.
- Bedroom area (BDA) shows the calculated fuzzy values that are grouped in the temporal window, to which the fuzzy temporal window membership function shown in Figure 4 has been applied, and that belong to beacons in the same bedroom area.
- Kitchen area (KTA) groups the calculated fuzzy values in the defined temporal window, as considered in Figure 4, and that belong to beacons located in the same kitchen area.
- Average Bathroom (Av BTA) shows the average value of the grouped values of the same bathroom area.
- Average Bedroom (Av BDA) represents the average value of the grouped fuzzy values of the same bedroom area.
- Average Kitchen (Av KTA) provides the average value of the grouped values of the same kitchen area.
- Location (Loc) is the location of the inhabitant in the smart lab based on the highest value of the averages obtained in each area.

The intelligent processing performed is based on the application of the equations presented in Section 3. The evaluation of these equations is detailed below.

To calculate the fuzzy value, we rely on the RSSI value read from the beacon. We use the fuzzy membership function illustrated in Figure 3 to obtain a proximity term between 0 and 1, giving more importance to RSSI values closer to the inhabitant, and reducing more distant RSSI values in order to distinguish the location of the inhabitant in the smart lab more clearly. Based on this fuzzy value, we calculate the proximity membership degrees considering its fuzzy temporal window defined in Figure 4. This temporal window gives preference to fuzzy values closer in time. This processing is described in Equation (1).

The next step in the processing involves applying Equation (2), clustering the previously obtained fuzzy values belonging to beacons located in the same area, taking into account the temporal window. The aggregation in each temporal window is presented in Table 2 in the column corresponding to each area (BTA, BDA and KTA).

For the aggregation of the fuzzy proximity terms of each area in a fuzzy temporal window, we apply Equation (3), where we obtain the average of fuzzy values for each area, as shown in the columns Average Bathroom (Av BTA), Average Bedroom (Av BDA) and Average Kitchen (Av KTA) in Table 2.

Finally, applying Equation (4), we calculate the location where the inhabitant is in the smart lab, obtaining the maximum of the previous aggregation values defined for each area. This information is illustrated in the Location column in Table 2.

### 4.3. Fuzzy vs. Non-Fuzzy Comparison

In this subsection, we compare the results obtained with the methodology using fuzzy processing versus non-fuzzy processing. To do so, a full day’s dataset is used, i.e., for the activities performed by the inhabitant in the morning, afternoon and evening.

Because the dataset is not labelled with the location but with the activity description, each proximity value was computed with the activity being carried out by the inhabitant according to the timestamp available in each data source. Furthermore, when there are proximity values to a beacon in the selected dataset but no specific activity is registered in that period of time, the activity value *Undefined* is assigned to that period.

**Figure 5:**
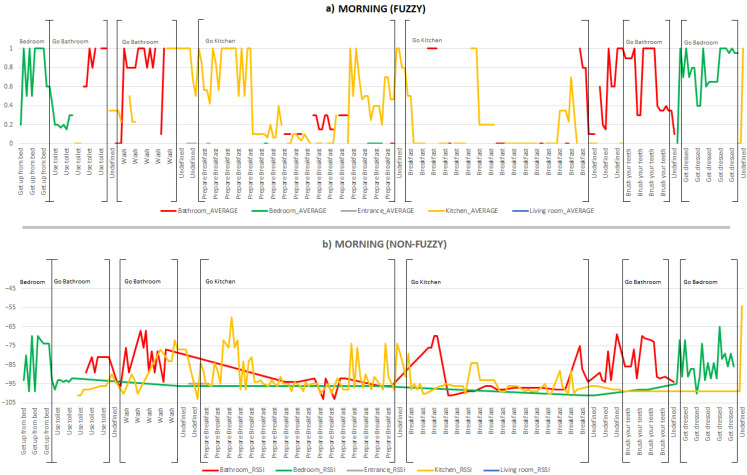
Areas visited and activities carried out by the inhabitant in the morning (**a**) with fuzzy processing and (**b**) non-fuzzy (raw data).

To describe the inhabitant’s location in the smart lab, we compare the location obtained with the proposed fuzzy processing and the location obtained without applying fuzzy processing, considering only raw RSSI values from the beacons provided by the UCAmI Cup dataset. The figures below represent the areas visited by the inhabitant in the morning, afternoon and evening, identifying each area of the smart lab with a different colour. There are two charts: Figure 5a (top) represents the visited areas applying the fuzzy processing described in the previous section, and Figure 5b (bottom) shows the areas visited by the inhabitant without applying any fuzzy processing. Furthermore, at the top of both charts, the areas of the smart lab involved in each activity are noted, based on the information shown in Table 1.

Figure 5 graphically represents the activities performed by the inhabitant in the morning, as well as the areas visited while performing them. The first of the morning activities carried out by the inhabitant is *Get up from bed*. During this activity, the inhabitant is in the bedroom area of the smart lab (green line). There is no significant difference in identifying the location of the inhabitant during this activity without applying fuzzy processing. Next, he/she performs the activity *Use toilet*. During this activity, we can observe in Figure 5a that the inhabitant goes to the bathroom area (red line) from the bedroom where he/she previously was in the smart lab (green line). In addition, there is a gap in the inhabitant’s path because in a short interval of time, weaker broadcasting signals are received from the kitchen beacons than from the bathroom. This is due to fluctuation in the broadcasting signals emitted by the beacons as well as in the frequency of emission. In Figure 5b, there is no gap, but it is more difficult to discern in which area the inhabitant is located, as similar values can be seen for different areas.

While the inhabitant performs the *Wash* activity, we observe in Figure 5a that the area in which the person is located must be the bathroom, although weaker signals from the kitchen are also represented, showing that with the processing the inhabitant’s location is correctly attributed to the bathroom (red line). At the end of this activity, we can observe that the processed data indicates that the inhabitant is in the kitchen area (yellow line). This inconsistency occurs due to the use of fuzzy temporal windows, as they can sometimes produce a displacement or delay when detecting a new area in which the inhabitant is located. If we observe Figure 5b, it is difficult to distinguish which area the inhabitant is in while performing the activity.

During the *Prepare breakfast* and *Breakfast* activities, the main area in which the inhabitant is located is the kitchen (yellow line), as can be seen in Figure 5a. However, during both activities, we identified moments where the inhabitant is recorded as being in the bathroom area, due to fluctuations in the broadcasting signals emitted by the beacons. At the end of the *Breakfast* activity, the inhabitant’s location is recorded as the bathroom area. This inconsistency is explained by the use of fuzzy temporal windows in the data processing carried out. Figure 5b shows situations in which the location of the inhabitant changes from one area to another. The fact of not giving preference to RSSI values that are closer in time and nearer the inhabitant means that there is a great variation in the detected location of the inhabitant and, therefore, a loss of precision when determining his/her real location.

Finally, the inhabitant performs the activity *Brush teeth* in the bathroom (red line) and *Get dressed* in the bedroom (green line). There are no significant differences between the data shown in Figure 5a,b for the location of the inhabitant in this period of time. We note again that Undefined represents moments when the inhabitant is in some area of the smart lab but no specific activity is identified, so we cannot determine which area the inhabitant is in during that period of time.

**Figure 6:**
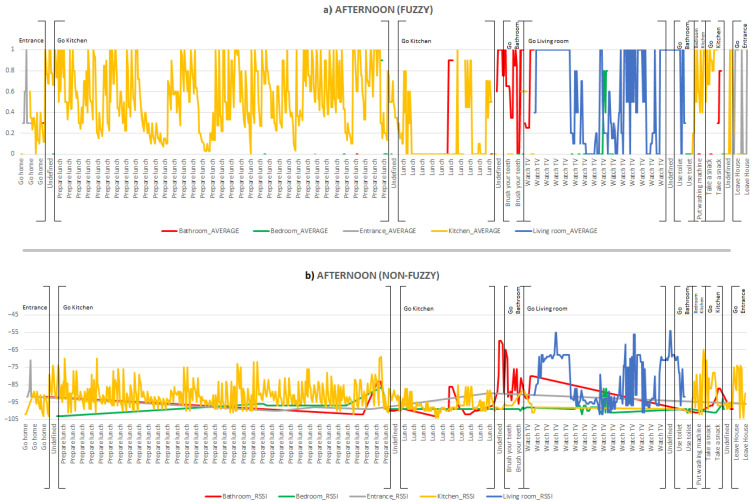
Areas visited and activities carried out by the inhabitant during the afternoon (**a**) with fuzzy processing and (**b**) non-fuzzy (raw data).

The activities carried out in the afternoon and the areas visited by the inhabitant in this period of time are illustrated in Figure 6. The first activity performed by the inhabitant is *Go home*, and the area where he/she is located is the entrance (grey line) in both Figure 6a,b, where no fuzzy processing is applied. Due to the use of temporal fuzzy windows in the processing, we can observe an inconsistency in the location data, as the inhabitant is located in the kitchen area at the end of the activity. Later, the inhabitant performs the activity *Prepare lunch* and Lunch, where the kitchen area (yellow line) predominates in both charts. However, it is in Figure 6a where the constant location of the inhabitant in the kitchen area can be observed, as the values of the kitchen area are given preference after applying fuzzy processing. While the inhabitant is performing the Lunch activity, there is a brief period where the processing locates the inhabitant in the bathroom area, which is caused by fluctuations in the broadcasting signals of the beacons. In Figure 6b, when carrying out the Lunch activity, there is greater variability in the location of the inhabitant, placing him/her in the kitchen, the bathroom or the entrance area.

Next, the inhabitant goes to the bathroom (red line) to perform the activity *Brush teeth*, and subsequently performs the activity *Watch TV* for a period of time in the living room (blue line).

Note that during this activity period, the inhabitant can be in several areas, as shown in Figure 6a, where there are moments in which the inhabitant goes from the living room to the bedroom, and then back to the living room. This is because the inhabitant may be moving between different areas when performing a particular activity, but one area predominates over the others. Again, in Figure 6b, there is no stable location of the inhabitant, showing that the inhabitant changes his/her location over short periods of time.

Then, for a short while, the inhabitant performs the activity *Use toilet*, but both charts show that the user is in the living room area. This is because of the values considered in this temporal window, as well as the short period of time in which this activity is carried out.

The inhabitant then carries out the *Turn on washing machine* activity. This activity involves the inhabitant going to the bedroom to get the laundry and then to the kitchen area to turn on the washing machine. Due to the short period of time in which the inhabitant performs the activity, the fuzzy processing performed does not detect values close to the bedroom area, showing only that the inhabitant is in the kitchen area (yellow line) while performing this activity, although the raw data in Figure 6b, show such values. The next activity is *Take a snack* where the inhabitant is located in the kitchen area (yellow line).

As with the activities carried out in the morning, there are periods of time during the afternoon when the activity is labelled as Undefined due to the inhabitant being in some area of the smart lab without performing a known specific activity. Finally, the inhabitant performs the activity *Leave house*. In Figure 6a, we can see that the inhabitant goes from the kitchen (yellow line) to the entrance area (grey line) to carry out this activity, but in Figure 6b, at no time is the inhabitant located at the entrance, but rather in the kitchen.

**Figure 7:**
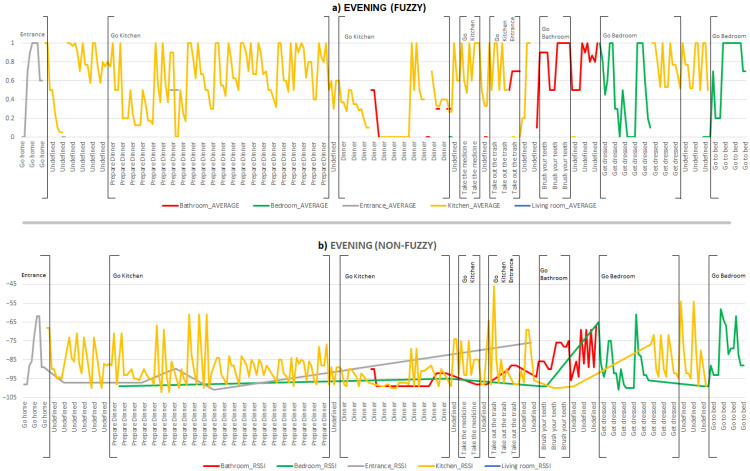
Areas visited and activities carried out by the inhabitant during the evening (**a**) with fuzzy processing and (**b**) non-fuzzy (raw data).

For the last time period described, activities carried out and areas visited in the evening are illustrated in Figure 7. The inhabitant performs the activity *Go home*, located in the entrance area of the smart lab. Both Figure 7a,b provide similar information for this activity. There is a period of time with Undefined activity, which indicates that the inhabitant is in some area but not performing a known activity. Next, the inhabitant performs the activities *Prepare Dinner*, Dinner and *Take medicine* in the kitchen area (yellow line). There is a greater stability in the location data shown in Figure 7a compared to the information provided in Figure 7b. However, we observe a small period in which the processing performed in Figure 7a incorrectly places the inhabitant in the bathroom. This is due to the data processed during that temporal window, where more data is processed from the bathroom area than from the kitchen where the inhabitant is really located. This can also be seen in Figure 7b where fuzzy processing has not been performed.

The next activity performed is *Take out the trash*, and as can be observed, in this activity the inhabitant has to go to the kitchen and then to the entrance of the smart lab. In this activity, Figure 7a has an inconsistency where the inhabitant is shown to be going to the bathroom (red line) instead of the entrance (grey line). Figure 7b shows that there is a delay and the inhabitant goes to the entrance once the *Take out the trash* activity is finished.

The inhabitant then carries out the *Brush your teeth* activity in the bathroom area (red line) and the *Get dressed* activity in the bedroom area (green line). While the inhabitant performs this activity, both Figure 7a,b show that the inhabitant finishes it in the kitchen area for a long period of time. This may be due to mislabelling of the dataset in the performance of the activity. Finally, the inhabitant performs the defined *Go to bed* activity in the bedroom area (green line).

To perform a qualitative comparison between the methodology using fuzzy logic and without using fuzzy logic, the fluctuations between areas are compared. Thus, the more the areas within an activity fluctuate, the lower the accuracy of the methodology. For example, if the activity “Brush teeth” is being performed, an accurate RSSI is one that always gives the location of the bathroom. In our case study, the bathroom beacons are close to the bedroom and the kitchen. Therefore, if the IPS, within the activity “Brush teeth”, computes bedroom and kitchen locations, it will be less accurate than an IPS that gives only the bathroom.

To qualitatively compute accuracy, each RSSI sample is determined by a beacon, which is assigned an area. In addition, this RSSI sample is assigned an activity, which is performed in one or more areas. Therefore, the ground truth is obtained from this information.

For each methodology (fuzzy logic and non-fuzzy logic), the location computed for each of the samples generated within each activity is compared. As shown in Table 3, for each of the samples within each activity, the number of True Positive (TP) is shown.

Table 3 shows that a higher accuracy is obtained when the location is computed by the fuzzy logic methodology by 10.51% percentage points.

## 5. UJAmI Location

In this section, we present UJAmI Location, which implements the fuzzy methodology presented in this paper. To do so, first, we offer a general description of the system, then we present its architecture and, finally, its functionality.

### 5.1. General Description

The indoor location system presented in this work is contextualised in the need to locate people with some kind of sensory, cognitive or mobility limitation in hospital buildings, care homes or residences. In such environments, it is very useful to know the location of the inhabitants in order to improve their care, detecting possible anomalies and improving resource management. For this reason, the system allows users to define basic elements, such as basic identification details (address, contact information, location map, etc.), as well as the different zones or areas into which the map is divided, the location of the beacons and the inhabitants of the space with their assigned devices. In this way, the system provides useful information on where inhabitants have been or are in real-time, how long they have been there and the most frequented areas in the space.

The designed system is called UJAmI Location, and it consists of a mobile application developed for Android operating system that searches for beacons inside a delimited space and sends the information to the server, as well as a web system that processes the information and manages the location data, both in real-time and over time, providing linguistic feedback to the user.

### 5.2. UJAmI Location Architecture

**Figure 8:**
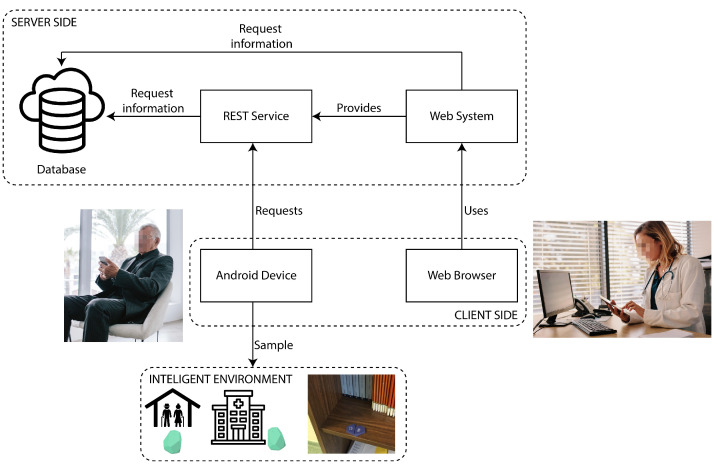
UJAmI Location architecture.

In this section, the architecture of the UJAmI Location system is presented. The aim of the system is to locate the inhabitant within any indoor environment in real-time and at all times. For this purpose, we have implemented a system that is based on the architecture shown in Figure 8.

In this architecture, we can distinguish three main components: sensors, client and server.

The sensors comprise Bluetooth beacons that are distributed among objects that are associated with areas of interest. For example, in a bedroom these objects can be the bed or the closets where clothes are stored. In this paper, Estimote Stickers (https://estimote.com/proximity, accessed on 5 August 2021) are used as BLE beacons integrated in the UJAmI Location, which were chosen due to their versatility. However, any other Bluetooth beacon can be integrated into the system.

On the client side, two elements can be highlighted: the mobile application, used for sample gathering and cloud storage, and the browser display, used to visualise the web system. The mobile application was developed for the Android operating system and was included in a mobile device that the inhabitant carried with him/her all the times. This application collects the samples and sends the RSSI values to be processed by the proposed method in the server. Furthermore, the website allows monitoring the inhabitant by visualising his or her information through the web browser of any conventional computer. By tracking through a website, we provide incredible versatility, ensuring full access to the service from a mobile device, a smart tablet or a computer, as long as there is an internet connection.

Finally, on the server side with the data model, three elements can be identified:

- The database with the model that is responsible for receiving, storing and retrieving the data.
- The REST service that acts as an intermediary between the database and any application that needs to store or retrieve information.
- The web service used to support the REST service and the web application to monitor the inhabitants.

The REST service provides a separation between the database and the client side, guaranteeing independence from the technologies and languages used, as well as high reliability, scalability and flexibility.

### 5.3. UJAmI Location Functionality

In this section, the functionality of UJAmI Location is presented.

**Figure 9:**
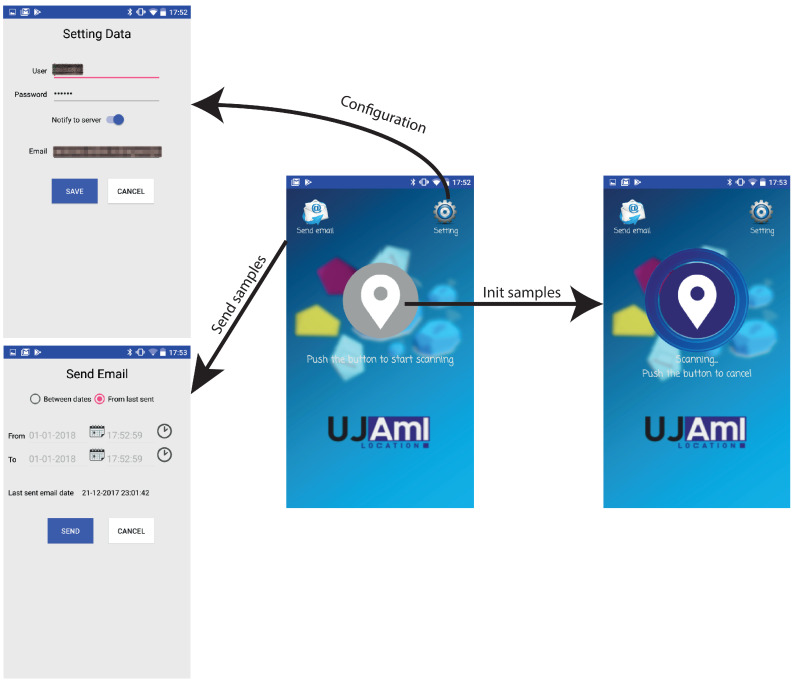
Mobile application interface.

As mentioned above, there are several essential components, but the main focus is on the mobile application and the monitoring web service. The main goal of the mobile application is to collect RSSI samples of the BLE beacons while inhabitants are in the smart environment, for example, inside a residence, a hospital or any enclosed place. The inhabitant wears a mobile device in which the application is installed to collect the samples automatically, process the information through the algorithm presented in the previous section and send the information to the database through the REST service. The interface of this application can be seen in the Figure 9.

Therefore, in summary, the information in the mobile application goes through the following steps:

1. Each inhabitant has an associated mobile device where the UJAmI Location application is started up.
2. The mobile device collects samples from RSSI beacons from a time frequency.
3. The mobile device processes the RSSI samples with the proposed fuzzy method in a fuzzy temporal window to compute the area where the inhabitant is according to the model presented in Section 3.
4. The mobile device sends the computed area to the server to store it.

Regarding the web service, it has been designed with the aim of providing information to the caregiver that is responsible for the inhabitant, whether in a nursing home or in a hospital.

**Figure 10:**
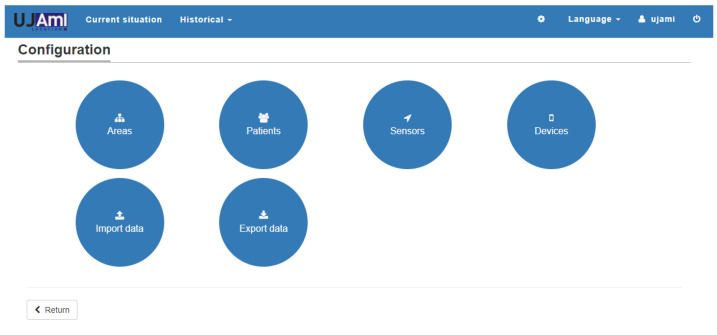
Management of different elements and data export and import.

**Figure 11:**
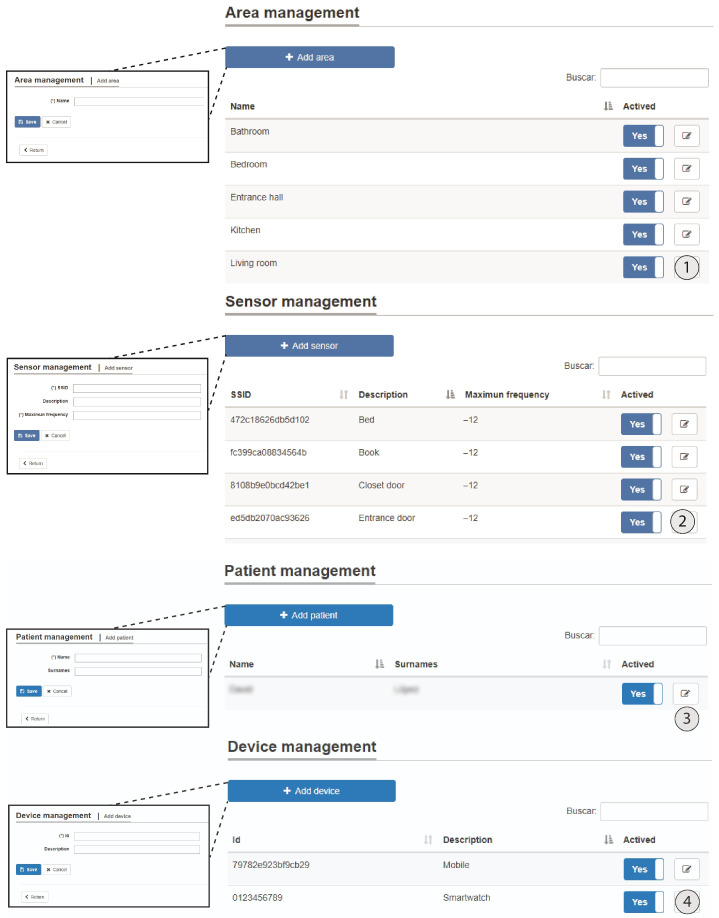
(**1**) Area management, (**2**) sensor management, (**3**) inhabitant management, and (**4**) device management.

From the admin role, the following functionality is provided: beacon management, user management and real-time monitoring of the inhabitant’s location in the areas that have been previously defined. Figure 10 and Figure 11 show the main screens of this monitoring system.

**Figure 12:**
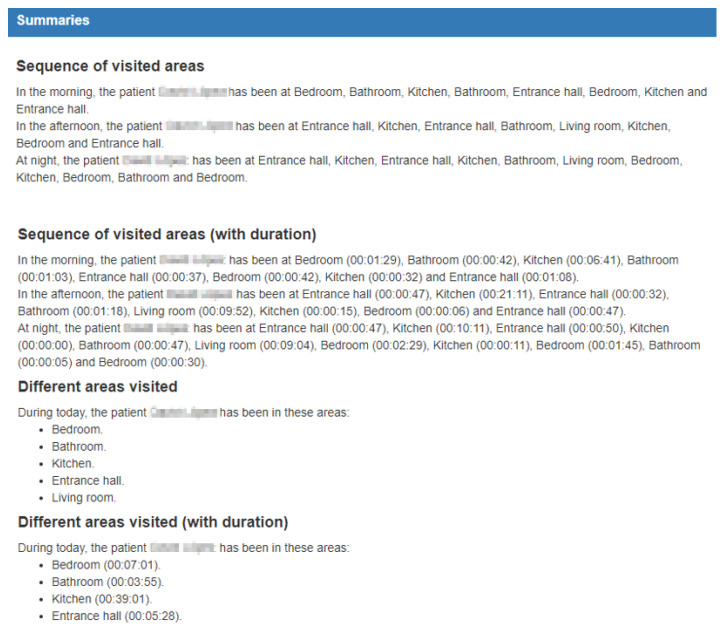
Summaries of the areas visited during a particular day in natural language.

**Figure 13:**
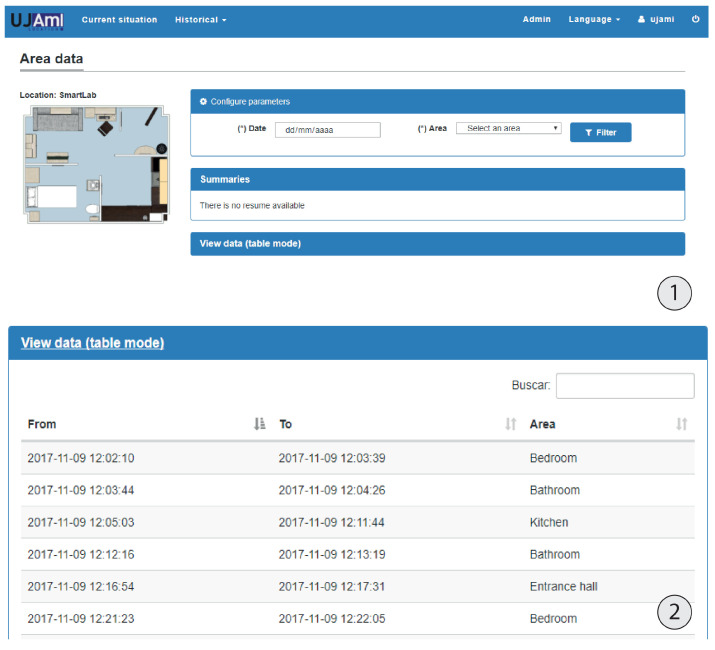
(**1**) Real-time tracking and (**2**) information about inhabitant location.

In addition, the system is not only capable of displaying the inhabitant’s location as shown in Figure 12, it is also capable of displaying a record of the inhabitant’s activity in natural language, showing a summary of what the inhabitant has done over a specific day. An example of this is illustrated in Figure 13.

## 6. Discussion

This section provides a discussion of the proposed theoretical methodology as well as the practical proposal, the UJAmI Location system, presented in this paper.

On the theoretical side, the fuzzy indoor location methodology has been validated with the UCAmI dataset, obtaining an accuracy of 91.63%, approximately 10 points higher than the methodology without using fuzzy logic. Regarding the strengths of our methodology, we point to the fact that it improves the identification of a person’s location within an indoor space while the inhabitant is performing a particular activity. The methodology succeeds in reducing variation in the location data produced by similar RSSI values in beacons located in different areas that do not correspond to the real location due to the nature of the signal emitted by the devices used. Giving preference to RSSI values that are closer in time and closer to the inhabitant provides increased stability in the inhabitant’s location and, therefore, higher accuracy. Thus, applying fuzzy processing on the data collected provides more stable and less variable location data of the person in fuzzy temporal windows.

Despite the advantages of the proposed methodology, we have also detected some limitations. The first one is that in short intervals of time, there are moments in which no signals are received from beacons located in the area where the inhabitant is, but broadcasting signals are received from beacons located in areas other than where the inhabitant is really located. This is due to fluctuations in the broadcasting signals emitted by the beacons, as well as in the frequency of emission. These problems were detected and mentioned in [48]. However, it is worth highlighting the successful performance of our theoretical proposal with fuzzy modelling, considering that the configuration of the beacons and power was preconfigured, so it was not configured and parameterised adhoc to achieve optimal results.

Another weakness of our proposal is that, at the end of some activities, the processing data indicate that the inhabitant is in a different area than the expected one. This inconsistency occurs due to the use of fuzzy temporal windows, as they can cause a delay when detecting a new area in which the inhabitant is located.

In the case study, where the inhabitant carries out an activity involving a single area over a long period of time, the inhabitant may be moving between different areas while performing this particular activity, but one area predominates over the others.

As far as our practical work, the UJAmI Location system, we discuss some important considerations such as security, privacy and deployment in a real environment. The proposed UJAmI Location system contains specific data regarding location and daily life, that is, information of a private nature. Security considerations need to be addressed in the mobile device [52]. In our case, the device only collects the RSSI values emitted by the beacons. These RSSI values contain noise, imprecision and fluctuations, requiring a methodology to compute the location correctly. The RSSI data are sent to the server by https protocol where the area or location of the person is computed. In our case, in a server on a web system with encrypted authentication where the RSSI data are received, processed and stored.

Regarding security and privacy, the location systems are no different in this regard to other systems based on IoT or based on information and communication technologies. It is necessary to deal with problems related to the authenticity, confidentiality, integrity and reliability of data exchanged with appropriate cryptography algorithms and robust security protocols [56].

Despite the risks that affect any ICT-based system, the UJAmI location system provides great benefits both to direct users (elderly people) and indirect users (family members and caregivers). The use of systems such as the one presented in this proposal can improve the quality of life, comfort and safety of the ageing population. In this regard, the proposal is aligned with the third goal of the UN’s Sustainable Development Goals entitled “Good health and well-being” [57,58].

## 7. Conclusions

A fuzzy indoor location methodology based on mobile devices and BLE beacons has been proposed in which RSSI streams are modelled according to a fuzzy linguistic approach to deal with the problem of uncertainty inherent to the use of BLE beacons. A case study has been presented in the UJAmI Smart Lab of the University of Jaén where the effectiveness of the proposed methodology is illustrated, comparing the processed results (91.63% accuracy) with results that have not been processed with fuzzy logic (81.12% accuracy). The proposed methodology has been integrated in a practical application, the UJAmI Location system. The architecture and the full functionality of this innovative system has been presented in the paper. It provides a key tool that to be used in the context of ageing populations. Our future works will focus on using data-driven approaches to train fuzzy classifiers to define the membership functions of the fuzzy linguistic variables involved.

## Figures and Tables

**Table 1:** Correspondence between activity and area.

ID Activity	Activity	Areas
Act01	Take medicine	Kitchen
Act02	Prepare breakfast	Kitchen
Act03	Prepare lunch	Kitchen
Act04	Prepare dinner	Kitchen
Act05	Breakfast	Kitchen
Act06	Lunch	Kitchen
Act07	Dinner	Kitchen
Act08	Take a snack	Kitchen
Act09	Watch TV	Living room
Act10	Go home	Entrance
Act11	Play a video game	Living room
Act12	Relax on the sofa	Living room
Act13	Leave house	Entrance
Act14	Visit in the smart lab	Entrance
Act15	Take out the trash	Kitchen, Entrance
Act16	Wash	Bathroom
Act17	Brush teeth	Bathroom
Act18	Use toilet	Bathroom
Act19	Wash dishes	Kitchen
Act20	Turn on washing machine	Bedroom, Kitchen
Act21	Work at the table	Workplace
Act22	Get dressed	Bedroom
Act23	Go to bed	Bedroom
Act24	Get up from bed	Bedroom
Act25	Read a book	Living room

**Table 2:** Subset of data corresponding to the *Brush teeth* activity.

TW	DT	Bc	Bc Area	RSSI	Fuzzy Value	BTA	BDA	KTA	Av BTA	Av BDA	Av KTA	Loc
1	13:29:29	BT	BTA	−89	0.6	[0.6]	-	-	0.6	-	-	BTA
2	13:29:29	TB	BTA	−88	0.7	[0.7, 0.6]	-	-	0.65	-	-	BTA
3	13:29:33	WB	KTA	−93	0.2	[0.35, 0.3]	-	[0.2]	0.35	-	0.2	BTA
4	13:29:33	PD	BDA	−92	0.3	[0.35, 0.3]	[0.3]	[0.2]	0.35	0.3	0.2	BTA
5	13:29:33	BT	BTA	−76	1.0	[1.0, 0.35, 0.3]	[0.3]	[0.2]	0.55	0.3	0.2	BTA
6	13:29:33	TB	BTA	−88	0.7	[0.7, 1.0, 0.35, 0.3]	[0.3]	[0.2]	0.59	0.3	0.2	BTA
7	13:29:39	BT	BTA	−76	1.0	[1.0]	-	-	1.0	-	-	BTA
8	13:29:39	PD	BDA	−94	0.1	[1.0]	[0.1]	-	1.0	0.1	-	BTA
9	13:29:39	TB	BTA	−88	0.7	[0.7, 1.0]	[0.1]	-	0.85	0.1	-	BTA
10	13:29:40	TB	BTA	−88	0.7	[0.7, 0.7, 1.0]	[0.1]	-	0.8	0.1	-	BTA
11	13:29:43	WD	BDA	−92	0.3	[0.7, 0.35, 0.5]	[0.3, 0.05]	-	0.52	0.17	-	BTA
12	13:29:44	PD	BDA	−100	0.0	[0.35, 0.0, 0.0]	[0.0, 0.3, 0.0]	-	0.12	0.1	-	BTA

TW: Temporal Window, DT: Datetime, Bc: Beacon, Bc Area: Beacon Area, BTA: Bathroom area, BDA: Bedroom area, KTA: Kitchen area, Av: Average, Loc: Location, WB: Water Bottle, MB: Medicine Box, BT: Bathroom Tap, TB: Toothbrush, PD: Pyjama Drawer.

**Table 3:** Qualitative comparison between the fuzzy and non-fuzzy processing methodologies.

Time of Day	Samples	Fuzzy TP	Fuzzy Accuracy	Non-Fuzzy TP	Non-Fuzzy Accuracy
Morning	219	193	88.13%	166	75.80%
Afternoon	773	712	92.11%	613	79.30%
Evening	226	211	93.36%	209	92.48%
Full-day	1218	1116	91.63%	988	81.12%

## Data Availability

All relevant data are within the paper and are available from the corresponding author. For a better visualisation of the key figures you can visit the following link: https://github.com/AntonioAlbin-dev/ujami_location, accessed on 5 August 2021.

## Abbreviations

The following abbreviations are used in this manuscript:

BLE	Bluetooth Low Energy
IoT	Internet of Things
IPS	Indoor Positioning Systems
RP	Reference Points
RSS	Radio Signal Strength
RSSI	Received Signal Strength Indicator
UJAmI Smart Lab	Smart Lab of the University of Jaén
